# Deoxycholic acid promotes development of gastroesophageal reflux disease and Barrett's oesophagus by modulating integrin‐αv trafficking

**DOI:** 10.1111/jcmm.13271

**Published:** 2017-09-22

**Authors:** David O. Prichard, Anne Marie Byrne, James O. Murphy, John V. Reynolds, Jacintha O'Sullivan, Ronan Feighery, Brendan Doyle, Osama Sharaf Eldin, Stephen P. Finn, Aoife Maguire, Deirdre Duff, Dermot P. Kelleher, Aideen Long

**Affiliations:** ^1^ Cell and Molecular Biology Group Department of Clinical Medicine Trinity Translational Medicine Institute Trinity College Dublin St James's Hospital Dublin 8 Ireland; ^2^ Division of Gastroenterology Mayo Clinic Health System La Crosse – Franciscan Healthcare La Crosse WI USA; ^3^ Division of Gastroenterology Mayo Clinic Rochester MN USA; ^4^ Department of Surgery Trinity Translational Medicine Institute Trinity College Dublin St James's Hospital Dublin 8 Ireland; ^5^ Department of Histopathology St James's Hospital Dublin 8 Ireland; ^6^ Department of Histopathology Beaumont Hospital Dublin 9 Ireland; ^7^ Faculty of Medicine Mansoura University Mansoura Egypt; ^8^ Department of Histopathology and Morbid Anatomy Trinity College Dublin Dublin Ireland; ^9^ Faculty of Medicine The University of British Columbia Vancouver BC Canada

**Keywords:** gastroesophageal reflux disease, Barrett's oesophagus, oesophageal adenocarcinoma, bile acids, integrin, cell adhesion molecule

## Abstract

The fundamental mechanisms underlying erosive oesophagitis and subsequent development of Barrett's oesophagus (BO) are poorly understood. Here, we investigated the contribution of specific components of the gastric refluxate on adhesion molecules involved in epithelial barrier maintenance. Cell line models of squamous epithelium (HET‐1A) and BO (QH) were used to examine the effects of bile acids on cell adhesion to extracellular matrix proteins (Collagen, laminin, vitronectin, fibronectin) and expression of integrin ligands (α_3_, α_4,_ α_5_, α_6_ and α_ν_). Experimental findings were validated in human explant oesophageal biopsies, a rat model of gastroesophageal reflux disease (GORD) and in patient tissue microarrays. The bile acid deoxycholic acid (DCA) specifically reduced adhesion of HET‐1A cells to vitronectin and reduced cell‐surface expression of integrin‐α_ν_ *via* effects on endocytic recycling processes. Increased expression of integrin‐α_v_ was observed in ulcerated tissue in a rat model of GORD and in oesophagitis and Barrett's intestinal metaplasia patient tissue compared to normal squamous epithelium. Increased expression of integrin‐α_ν_ was observed in QH BO cells compared to HET‐1A cells. QH cells were resistant to DCA‐mediated loss of adhesion and reduction in cell‐surface expression of integrin‐α_ν_. We demonstrated that a specific component of the gastric refluxate, DCA, affects the epithelial barrier through modulation of integrin α_ν_ expression, providing a novel mechanism for bile acid‐mediated erosion of oesophageal squamous epithelium and promotion of BO. Strategies aimed at preventing bile acid‐mediated erosion should be considered in the clinical management of patients with GORD.

## Introduction

Erosion of oesophageal squamous epithelium induced by gastroesophageal reflux disease (GORD) is associated with an increased risk of developing Barrett's oesophagus (BO). This premalignant condition underlies the majority of oesophageal adenocarcinoma 1, 2. Low pH and bile acids in oesophageal refluxate both increase the risk of epithelial erosion and are independent risk factors for the development of BO 3, 4. Cell death was thought to be the mechanism through which these agents drove erosive oesophagitis. However, the identification of dilated intercellular spaces within oesophageal epithelium occurring in response to refluxate and resolving after GORD treatment suggested that intercellular adhesion is being disrupted in response to GORD, reducing cellular adhesive strength and impairing pro‐survival signalling 4, 5, 6, 7, 8.

Alterations in tight junction proteins in response to GORD or its constituents have now been well documented. In animal models of GORD, relocalization of claudin‐4 and occludin from the plasma membrane to the cytoplasm in the spinous and granular layers of oesophageal epithelium is observed in response to reflux 9. Human tissue explant studies demonstrate up‐regulated gene and protein expression of the tight junction proteins claudin‐1 and 2, but not occludin or zona occludens‐1 or 2, in patients with erosive reflux disease 10. Similarly, reduced expression of E‐cadherin has been demonstrated in response to reflux 11. *In vitro* research suggests that the localization of claudin‐4 to tight junction complexes is disrupted by exposure to low pH 12. The unconjugated bile acid deoxycholic acid (DCA) at neutral pH impairs epithelial function and alters the localization of claudin‐1, claudin‐4 and E‐cadherin 13, 14. Moreover, bile acids and low pH appear to act synergistically to alter epithelial barrier function 13, 15. However, intercellular adhesion is additionally mediated by molecules other than tight junction proteins and tight junctions do not mediate adherence between cells and the extracellular tissue scaffolding.

Cellular adhesion to extracellular matrix (ECM) proteins is primarily mediated through hetero‐dimeric proteins called integrins 8. Comprised of one α‐ and one β‐subunit, integrins bind with variable affinity and avidity to specific ECM proteins to provide anchorage and activate pro‐survival signalling 8. Intercellular adhesion mediated by integrins has also been described in squamous epithelium 16, 17, and the presence of integrin‐α_2_, α_3_, α_6_ and α_v_ has been demonstrated in oesophageal squamous epithelium 18, 19, 20. These adhesion molecules are constantly recycled in order to facilitate tissue remodelling in response to physiological stress. Insufficient integrin‐ligand binding can result in reduced adhesive strength, detachment of cells from the ECM and, due to the absence of appropriate survival signalling, apoptosis 21, 22, 23.

In this study, we investigated how a specific component of the gastric refluxate, DCA, affects the epithelial barrier through modulation of integrin expression, providing a novel mechanism for bile acid‐mediated erosion of oesophageal squamous epithelium and facilitating re‐epithelialisation with BO.

## Materials and methods

### Cell lines and culture

HET‐1A and QH‐Tert (also known as CP‐A) 24, 25 cell lines, representing oesophageal squamous epithelium and non‐dysplastic metaplasia (BO), respectively, were used for these experiments and cultured according to manufacturer's instructions (ATCC, Manassas, VA, USA).

### Adherence and detachment assays

Adhesion Assays: Detached HET‐1A cells were seeded in 96‐well plates. Simultaneously 100 μl of medium containing DCA or ursodeoxycholic acid (UDCA; Sigma‐Aldrich, St. Louis, MO, USA) was added to each well. After allowing 2 hrs for adhesion, the medium was aspirated, the cells washed, and 100 μl of medium containing 2.5 μM calcein AM (Biotium, Hayward, CA, USA) was added to each well for 1 hr at 37°C. Fluorescence was determined using a Victor luminometer (Perkin Elmer, Waltham, MA, USA). The Millicoat™ ECM screening kit (Millipore, Billerica, MA, USA) was used to determine adhesion to specific ECM proteins.

Detachment and Re‐Adherence Assays: cells were seeded in 12‐well plates and allowed to adhere overnight. After 2 hrs treatment with DCA, the growth medium was aspirated and the wells washed twice with medium to ensure capture of all detached cells. Detached cells were re‐suspended in fresh medium and placed in a new well. Wells containing the residual adherent cells were washed twice, and fresh medium was added to each well. After 24 hrs, images were acquired and cell viability determined using MTT (Sigma‐Aldrich, St. Louis, MO, USA). The original untreated well was used as the reference for comparison.

### Flow cytometric assessment of integrin expression

Flow cytometry was used to investigate cell‐surface integrin expression using anti‐integrin antibodies (α_3_/α_4_/α_5_/α_6_; BD Biosciences, Franklin Lakes, NJ, USA, α_v_; Merck, Whitehouse Station, NJ, USA, and Santa Cruz Biotechnology Inc, Santa Cruz, CA, USA) with Alexa Fluor secondary antibodies (Invitrogen, Carlsbad, CA, USA). Fluorescence was determined using a Beckman Coulter flow cytometer (Cyan ADP 9 Colour, Brea, CA, USA).

### Western blot analysis

Western blot procedure was performed as described previously 26 using anti‐integrin‐α_v_ (BD Biosciences, Franklin Lakes, NJ, USA) and anti‐β‐actin (Sigma‐Aldrich, St. Louis, MO, USA).

### Immunofluorescence microscopy

HET‐1A cells were fixed with 4% paraformaldehyde. Expression of integrin‐α_v_ and Rab11 was detected using primary antibodies (integrin‐α_v_: BD Biosciences, Rab11; Zymed Laboratories, San Francisco, California, United States) and appropriate secondary antibodies (Alexa Fluor; Invitrogen, Carlsbad, CA, USA). Nuclei were stained with Hoechst 44432 (Invitrogen, Carlsbad, CA, USA). Images were acquired using the IN Cell 1000 (GE Healthcare, Waukesha, WI, USA) or Zeiss LSM510 laser confocal microscope (Carl Zeiss, Oberkochen, Germany). Image analysis was performed using IN Cell analysis software.

### *Ex vivo* oesophageal tissue biopsy culture

For explant experiments, biopsies of healthy squamous oesophageal tissue were acquired with patient's consent, during gastroscopy in the endoscopy unit of St James's Hospital, Dublin 8, Ireland. No patients had symptoms or macroscopic evidence of GORD. Six biopsies were taken from each of five patients, 5 cm above the gastroesophageal junction. Following incubation with DCA for 2 hrs, tissue explants were formalin fixed and embedded in paraffin blocks for analysis of integrin‐α_v_ expression by immunofluorescence. After deparaffinization and epitope retrieval, the tissue was sequentially incubated with primary (anti‐α_v_), and secondary antibodies and Hoechst as above. Images were acquired using a Zeiss LSM510 laser confocal microscope using a 63× oil immersion objective lens.

### Quantification of intensity of membrane staining with integrin α_v_ in tissue explants after DCA treatment

Tissue explants were acquired, treated, fixed, stained and imaged as described above. The Trainable Weka Segmentation Plugin (available at http://fiji.sc/Trainable_Weka_Segmentation) for ImageJ (US National Institutes of Health, Bethesda, MD, USA) was used to define the cell membranes prior to determining membrane intensity (Fig. S2).

### Immunohistochemistry of patient oesophageal tissue microarrays

Tissue microarrays (TMA) were constructed from diagnostic blocks of oesophageal disease at St James's Hospital. Case selection and approval of TMAs were conducted (JOS). Areas of disease were identified by a consultant pathologists (BD, OSE), and 0.6 mm cores were taken from the blocks to construct the TMAs (RF). Pathology was re‐evaluated post‐TMA construction (BD, OSE). Normal control samples were obtained from individuals attending for upper GI endoscopy without symptoms to suggest GORD or other inflammatory conditions and whom upon pathological assessment demonstrated no evidence of inflammation or disease. Immunohistochemistry was performed on normal squamous epithelium (*n* = 22), oesophagitis (*n* = 52) and Barrett's intestinal metaplasia (IM; *n* = 70) tissue using anti‐integrin‐α_v_ and the Vectastain Elite ABC HRP Kit (Vector Laboratories, Peterborough, United Kingdom) according to manufacturer's instructions. Tissue microarrays were imaged using the Aperio Digital pathology slidescanner (Leica Biosystems, Nussloch, Germany). Immunoreactivity was assessed digitally under 40× magnification in a semi‐quantitative manner for integrin‐α_v_ by two independent observers (AMB and DD) who were blinded to the pathological and clinical diagnosis of all patients in the study. Both percentage positivity and of cytoplasmic staining intensity were graded for epithelial and stromal compartments. Intensity was graded as 0 (negative), 1 (weak), 2 (moderate) and 3 (strong), and positivity was graded as 0%, 25%, 50%, 75%, or 100%.

### Animal model of GORD

Oesophageal tissue for this study was obtained as per a previous study 27. Briefly, oesophagojejunostomies were performed on Wistar rats in order to promote mixed acid and bile acid reflux. At 22 ± 2 weeks, the animals were sacrificed, the oesophagus was removed, fixed in 10% buffered formalin, processed and paraffin‐embedded. A control group (*n* = 4) who did not receive oesophagojejunostomy were also included. Tissue sections were stained with haematoxylin and eosin, and areas of ulcerated and non‐ulcerated tissue were identified by consultant pathologist (SF) who was blinded to experimental grouping. Immunohistochemical staining of tissue for integrin‐α_v_ was carried out as described above for quantification purposes (*n* = 4 control group, *n* = 12 from oesophagojejunostomy group). Images were acquired using an Aperio Digital pathology slidescanner (Leica Biosystems). Staining intensity of integrin‐α_v_ was assessed digitally under a 10× objective, in a semi‐quantitative manner by two independent observers (AMB, DD) as above. Analysis of staining intensity in ulcerated tissue was compared to non‐ulcerated tissue for each animal.

### Statistics

Data were analysed using Graph Pad Prism (Graph Pad Prism, San Diego, CA, USA). Data are presented at mean ± S.E.M. for *n* = 3 experiments. Data were analysed using one‐way ANOVA, *t*‐tests, Kruskal–Wallis tests or Mann–Whitney *U*‐tests as appropriate. Differences with *P*‐values <0.05 (*), <0.01 (**) and <0.001 (***) were considered statistically significant.

### Ethical approval

Ethical approval to conduct the work with the patient tissue was granted by the Adelaide and Meath Hospital (AMNCH), Tallaght, Dublin (REC 2011/04/05). Ethical approval for the rat GORD model was granted by the BioEthics Committee, Trinity College Dublin, Ireland.

## Results

### DCA reduces adhesion and promotes detachment of HET‐1A cells

**Figure 1:**
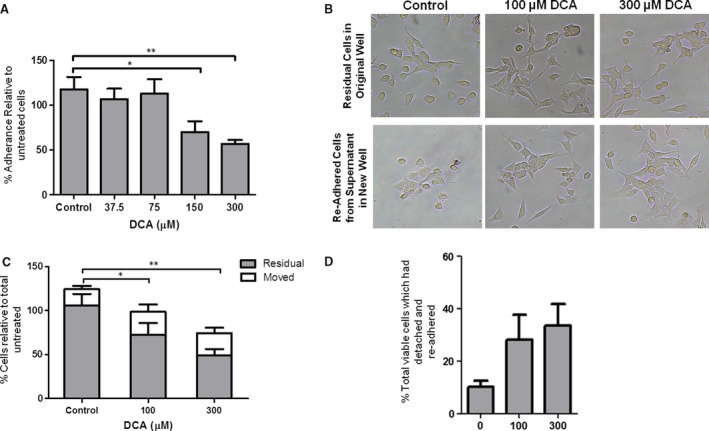
DCA‐mediated effects on cell adhesion. (**A**) DCA impairs adhesion of HET‐1A cells. Cultured cells were mechanically detached by gentle scraping and seeded in a fresh well with growth medium containing DCA for 2 hrs. DCA was removed, the cells incubated with calcein AM and fluorescence determined. Adherence was normalized to control. (**B**–**D**) DCA causes detachment of adherent HET‐1A cells. Adherent HET‐1A cells were exposed to DCA for 2 hrs. The residual adherent cells were washed to remove DCA and fresh growth medium added. Detached cells, present in the growth medium used for DCA stimulation, were centrifuged and washed to remove DCA prior to re‐seeding in a new culture well. After 24 hrs, images were acquired to document re‐adherence of the detached cells (**B**). Cellular viability in each well, relative to untreated unmoved cells, was determined by MTT assay (**C**) and used to determine and the percentage of viable cells which had detached/re‐adhered in response to DCA exposure (**D**). Results are presented as mean and S.E.M. for *n* = 3 experiments (*P < 0.05, ***P < 0.001 relative to untreated cells).

To identify whether bile acids affected oesophageal cell adhesion, we exposed HET‐1A cells to DCA or UDCA during the process of adhesion. HET‐1A cells were seeded in the presence of DCA or UDCA for 2 hrs. Deoxycholic acid prevented adhesion in a dose‐dependent fashion, reducing adherence to 70.2% (*P* < 0.05) and 57.0% (*P* < 0.001) of control at concentrations of 150 and 300 μM, respectively (Fig. 1A). The hydrophilic bile acid UDCA had no effect (Fig. S3). Neither DCA nor UDCA affected cell viability or induced apoptosis at these concentrations and time‐points (Fig. S4A and B, respectively). The concentrations chosen were based on reported concentrations found present in refluxate from patients with GORD and BO 28.

We next investigated whether DCA caused detachment of adherent HET‐1A cells. After 2‐hr exposure to DCA, the detached cells in the supernatant were aspirated, washed to remove the bile acid stimulus and re‐seeded into fresh 12‐well plates. Detachment, and re‐adherence, of 28.1% and 33.6% of viable cells was observed in response to 100 and 300 μM DCA, respectively (*P* < 0.001, Fig. 1B–D). These findings suggest that exposure to DCA induces a loss of cell adhesion which is reversible and is independent of effects on cell viability. This is a dose‐dependent effect, as 500 μM DCA, although not representative of physiological concentrations, caused detachment of the cells and a subset of the detached cells are still viable and re‐adhere (Fig. S1).

### DCA reduces adhesion to a specific subset of ECM proteins

**Figure 2:**
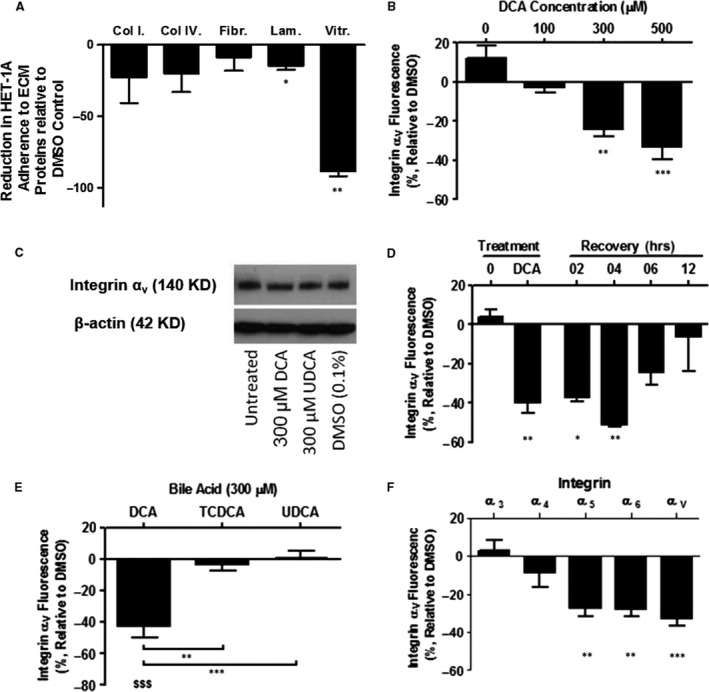
DCA stimulation induces a reversible reduction in the cell‐surface expression of a select cohort of integrins.. Adhering HET‐1A cells were stimulated for 2 hrs with bile acids. The number of cells attached to the specific ECM protein after treatment was determined by measuring absorbance of dissolved crystal violet at 570 nM. Abbreviations: Col I: collagen I, Col IV: collagen IV, Fibr: fibronectin, Lam: laminin, Vitr: vitronectin (**A**). DCA stimulation results in a dose‐dependent reduction in the cell‐surface expression of integrin‐α_v_ (**B**) without altering total cellular protein expression (**C**). This reduction of cell‐surface integrin‐α_v_ expression was reversible after withdrawal of the DCA stimulus (**D**). The cell‐surface expression of integrin‐α_v_ is not affected by UDCA or TCDCA (**E**). The reduction in cell‐surface expression is limited to a select cohort of integrins (**F**). Fluorescence was determined by flow cytometry. Results are presented as mean and S.E.M. for *n* = 3 experiments. (*P < 0.05, **P < 0.01 and ***P < 0.001 relative to DMSO control).

To determine whether the loss of adhesion induced by DCA was a general or selective phenomenon, adherence to specific ECM proteins was assessed. In response to 300 μM DCA treatment, there was no alteration in adherence of HET‐1A cells to collagen I, collagen IV or fibronectin relative to DMSO control. However, a significant reduction in adhesion to vitronectin (88.6%, *P* < 0.01, Fig. 2A) and laminin (14.6%, *P* < 0.05, Fig. 2A) was observed.

### DCA reduces the surface expression of a select group of integrins

Adhesion to vitronectin is almost exclusively mediated through integrin‐α_v_ 29. Therefore, we next investigated whether the reduction in adhesion was a consequence of altered cell‐surface integrin expression. Expression of integrin‐α_v_ was reduced by 2.8%, 24.2% (*P* < 0.01) and 33.2% (*P* < 0.001) in response to 100, 300 and 500 μM DCA, respectively (Fig. 2B) demonstrating a dose‐dependent effect. These findings were replicated using an antibody directed against an alternate epitope on the extracellular domain of integrin‐α_v_ (17E6 clone, Merck, data not shown) excluding a conformational shape change as the cause. Additionally, in order to exclude a processing artefact due to the mechanical detachment process used (cell scraping), immunofluorescent staining of adherent HET‐1A cells exposed to DCA was performed, imaged using high content analysis and assessed using IN Cell Investigator software. In response to DCA, a reduction in cell membrane integrin‐α_v_ expression was observed with no change in total integrin‐α_v_ expression (Fig. S5). Similarly, Western blotting demonstrated that DCA treatment had no effect on total cellular integrin‐α_v_ protein expression (Fig. 2C). These findings suggest that DCA mediates detachment of HET‐1A cells from vitronectin by reducing the cell‐surface expression of integrin‐α_v_ rather than reducing protein expression. In view of the observed ability of HET‐1A cells to re‐adhere after removal of DCA stimulus, we next evaluated whether the reduction in cell‐surface integrin expression was a reversible phenomenon. Following withdrawal of DCA, the cell‐surface expression of integrin‐α_v_ returned to baseline levels (Fig. 2D). Finally, in order to evaluate whether other components of oesophageal refluxate exhibited a similar effect on integrin expression, HET‐1A cells were exposed to the conjugated bile acid taurochenodeoxycholic acid (TCDCA) and the hydrophilic bile acid UDCA. Neither TCDCA nor UDCA induced a change in the cell‐surface expression of integrin‐α_v_ (Fig. 2E) suggesting that, among the bile acids studied in these experiments, the effects on integrin‐α_v_ internalisation are specific to DCA. The effect of DCA on a cohort of integrins was then investigated to examine whether this was a generalized process affecting integrin expression. Deoxycholic acid (300 μM) exposure reduced the cell‐surface expression of integrins‐α_5_, α_6_ and α_v_ by 29.3% (*P* < 0.01), 28.1% (*P* < 0.01) and 32.6% (*P* < 0.001), respectively, relative to control (Fig. 2F). The cell‐surface expression of integrins‐α_3_ and α_4_ was not significantly altered (Fig. 2F). These results suggest that exposure of HET‐1A squamous epithelial cells to DCA induces a reversible reduction in the cell‐surface expression of a select cohort of integrins.

### The membrane expression of integrin‐α_v_ is reduced by DCA in oesophageal tissue explants

**Figure 3:**
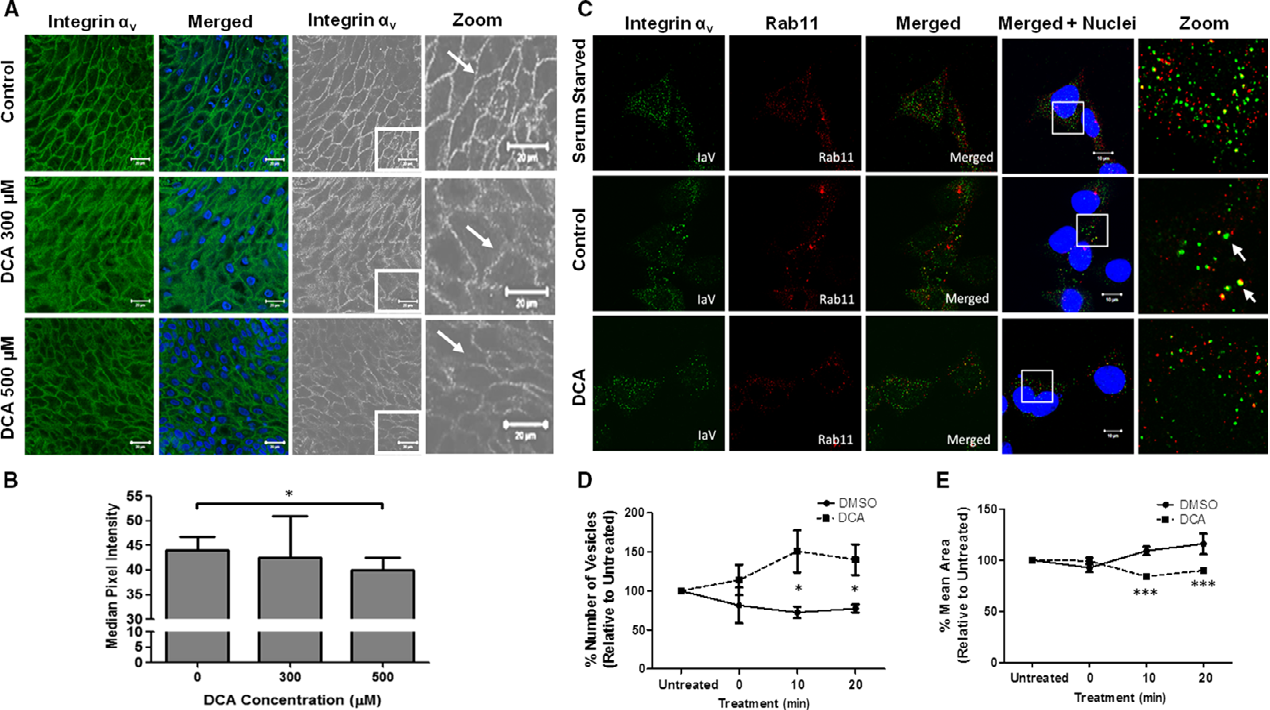
(**A** and **B**) DCA reduces integrin‐α_v_ expression in oesophageal tissue explants. Tissue explants (*n* = 5 per group) were exposed to DCA *ex vivo* for 2 hrs, fixed, paraffin‐embedded and subsequently stained for integrin‐α_v_ expression. Images were acquired using a Zeiss 510 confocal microscope (**A**). Membranes and membrane intensity were identified using the Trainable Weka Segmentation Plugin in ImageJ (**B**, see Fig. S2). (**C**–**E**) DCA impairs integrin‐α_v_ endocytic recycling. To inhibit endocytic recycling, HET‐1A cells were serum starved. No co‐localization between Rab11 and integrin‐α_v_ was observed (**C**, serum starved, top panel). Complete medium was then added to allow endocytic recycling to progress, as indicated by fusion of vesicles to form larger vesicles and co‐localization of Rab11 and integrin‐α_v_ (**C**, control, middle panel, arrows). When HET‐1A cells were exposed to complete medium containing DCA, no fusion of vesicles or co‐localization between Rab11 and integrin‐α_v_ was observed (**C**, DCA, bottom panel). The mean number of vesicles (**D**) and mean area of vesicles (**E**) were imaged by high content analysis and quantified using the IN Cell Investigator software package. Data are presented as mean ± S.E.M. for DCA treatment relative to DMSO control for *n* = 3 experiments (*P < 0.05, ***P < 0.001).

In order to determine whether the *in vitro* findings were representative of an *in vivo* phenomenon, we used an *ex vivo* tissue explant model. Integrin‐α_v_ was localized to the cell membrane in oesophageal explant biopsy tissue (Fig. 3A, arrows). Quantification of membrane intensity after exposure of the explanted tissue to DCA (Fig. S2) demonstrated that the mean membrane fluorescent intensity of integrin‐α_v_ was reduced by exposure to 500 μM DCA (*P* < 0.05, Fig. 3B). Although we observed more diffuse expression of integrin‐α_v_, particularly in biopsies treated with 300 μM DCA, we were unable to quantify this due to limitations in sensitivity using current image analysis software programs. The data suggest that the observations detected *in vitro* are recapitulated in human patient *ex vivo* treated tissue.

### DCA impairs endosomal processing

The observed reduction in surface expression of integrin‐α_v_ (Fig. 2B) suggested protein internalization. The type of pathway through which internalized proteins/receptors progress can be identified by their association with specific Rab‐GTPases. The co‐localization of integrin‐α_v_ with Rab11 in HET‐1A cells suggests processing through a recycling pathway rather than internalization for degradation (Fig. 3C arrows) 29. Progression of endosomes through the recycling pathway is associated with fusion of endosomal vesicles, resulting in fewer Rab‐associated vesicles of larger size 30. After serum starvation, HET‐1A cells exposed to complete medium with DMSO (vehicle control) demonstrated a reduction in the number of Rab11 vesicles (Fig. 3C middle panel and D) and an increase in mean vesicular size (Fig. 3E) consistent with reactivation of endocytic recycling. Deoxycholic acid exposure resulted in a greater number of smaller Rab11‐positive vesicles relative to control (Fig. 3C bottom panel, D and E). This suggests that when cells are exposed to DCA, endosomes are failing to fuse and mature into larger endosomes and are thus not progressing through the endocytic recycling pathway 30. This results in reduced protein recycling and cell‐surface expression of integrin‐α_v._

### The expression of integrin‐α_v_ is increased in ulcerated tissue in an animal model of GORD

**Figure 4:**
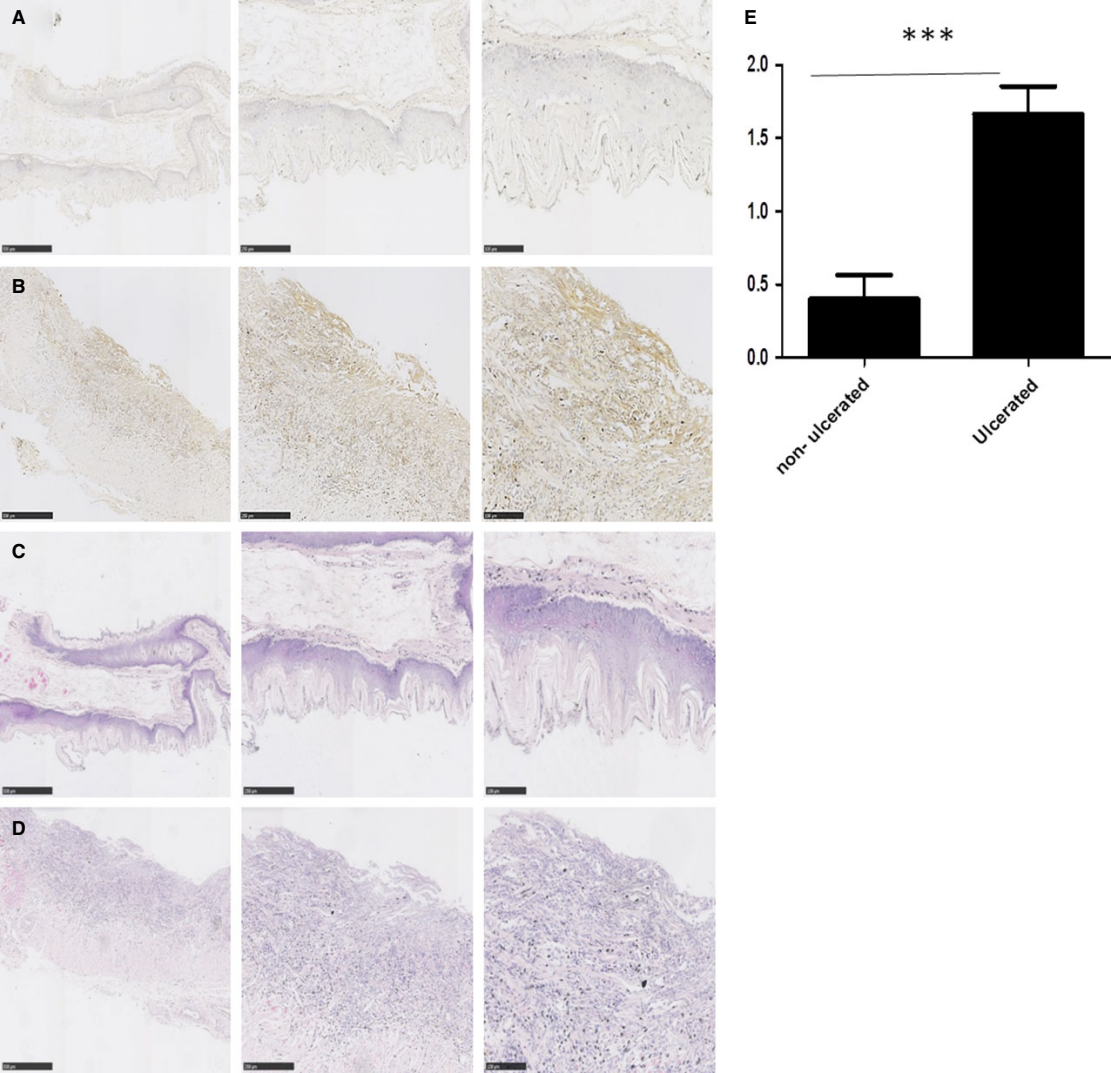
Integrin‐α_v_ expression is increased in ulcerated tissue compared to non‐ulcerated squamous epithelium in an *in vivo* model of GORD. Oesophageal specimens from rats (*n* = 12) exposed to pathological reflux were examined for expression of integrin‐α_v_. A significant increase in integrin‐α_v_ expression was observed in the ulcerated regions (**B**) compared to normal non‐ulcerated squamous epithelium (**A**) (***P < 0.001 Mann–Whitney *U*). **C** and **D** are the corresponding haematoxylin and eosin stains for **A** and **B**, respectively.

Chronic exposure of the oesophagus to bile and acid results in pre‐metaplastic changes and formation of ulcers. We used a rat model of GORD to investigate the effect of chronic exposure of refluxate on the expression of integrin‐α_v_. Oesophageal specimens from rats (*n* = 12) which had been exposed to pathological reflux were examined for expression of integrin‐α_v_ (Fig. 4). A significant increase in integrin‐α_v_ expression was observed in the adherent epithelium adjacent to the ulcerated regions (Fig. 4B) compared to normal non‐ulcerated squamous epithelium (Fig. 4A). These results demonstrate that chronic exposure of the oesophageal squamous epithelium to refluxate leads to pre‐metaplastic changes in integrin‐α_v_ expression, suggesting that up‐regulation of integrin‐α_v_ expression is an early event occurring in erosive GORD, prior to the development of IM.

### Integrin‐α_v_ expression is up‐regulated in oesophagitis and Barrett's IM patient tissue

**Figure 5:**
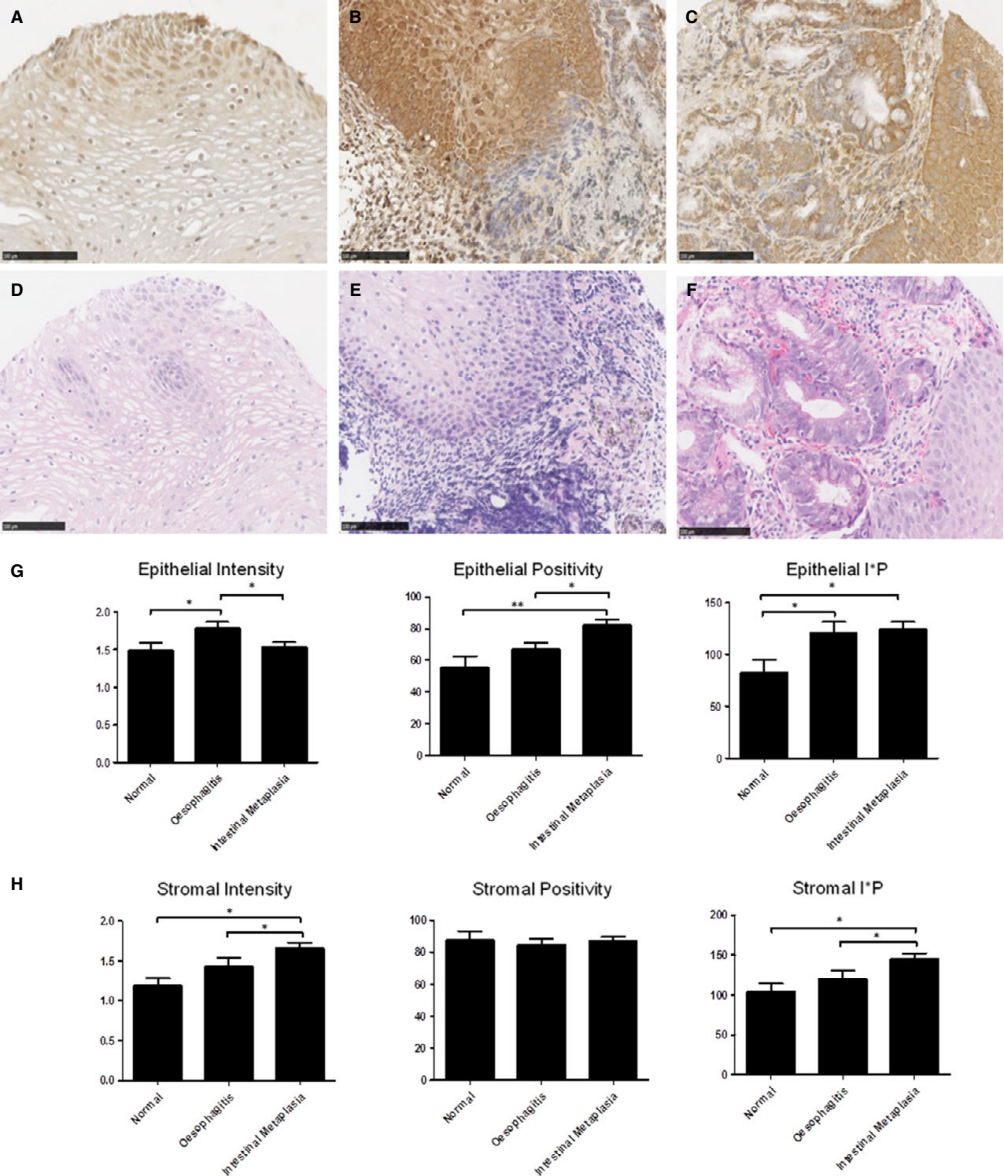
Integrin‐α_v_ expression in patient tissue. Images of integrin‐α_v_ staining of normal (**A**) oesophagitis (**B**) and Barrett's IM (**C**) together with corresponding haematoxylin and eosin staining of the same tissue (**D**–**F**). (**G**) A significant increase in integrin‐α_v_ epithelial expression (Intensity) was observed between normal and oesophagitis (*P < 0.05, Mann–Whitney *U*) and between oesophagitis and IM (*P < 0.05, Mann–Whitney *U*). A significant increase in the percentage of positively stained epithelial cells was observed between normal and IM (**P < 0.01, Kruskal–Wallis, ***P < 0.001 Mann–Whitney *U*) and between oesophagitis and IM (*P < 0.05 Kruskal–Wallis, **P < 0.01 Mann–Whitney *U*). A significant increase in epithelial I*P was observed between normal and oesophagitis (*P < 0.05, Mann–Whitney *U*) and between normal and IM (*P < 0.05 Kruskal–Wallis, **P < 0.01 Mann–Whitney *U*). (**H**) A significant increase in stromal integrin‐α_v_ expression (Intensity) was observed between normal and IM (*P < 0.05 Kruskal–Wallis, **P < 0.01 Mann–Whitney *U*) and between oesophagitis and IM (*P < 0.05 Kruskal–Wallis, *P < 0.05 Mann–Whitney *U*). No differences were observed in the percentage of positive stromal staining for integrin‐α_v_ between normal, oesophagitis and IM. A significant increase in stromal I*P was observed between normal and IM (*P < 0.05 Mann–Whitney *U*) and between oesophagitis and IM (*P < 0.05 Kruskal–Wallis, *P < 0.05 Mann–Whitney *U*). *P*‐values <0.05 (*), <0.01 (**) and <0.001 (***).

To assess integrin‐α_v_ expression in patient tissue, we performed immunohistochemical analysis using tissues from the following patient groups: normal squamous epithelium (*n* = 22), oesophagitis (*n* = 52) and Barrett's IM (*n* = 70). Figure 5 shows representative images of integrin‐α_v_ in tissue from normal squamous epithelium (Fig. 5A), oesophagitis (Fig. 5B) and IM (Fig. 5C) together with their corresponding tissue sections stained with haematoxylin and eosin (Fig. 5D–F). We observed an increase in integrin‐α_v_ expression in the epithelium of oesophagitis tissue in terms of epithelial intensity, compared to normal squamous epithelium or Barrett's IM (Fig. 5G, both *P* < 0.05). A significant increase in the number of positively stained epithelial cells was observed in IM tissue compared with normal squamous epithelium (Fig. 5G, *P* < 0.01) or oesophagitis tissue (Fig. 5G, *P* < 0.05). Using a combined score, incorporating both epithelial intensity and epithelial positivity demonstrated an increase in integrin‐α_v_ in oesophagitis and IM tissue compared to normal squamous epithelium (Fig. 5G, both *P* < 0.05). We also examined the expression of integrin‐α_v_ in the stromal compartment for the different tissue subsets. An increase in protein expression (intensity) was observed in IM tissue compared to normal squamous epithelium or oesophagitis tissue (Fig. 5H, *P* < 0.05). There was no difference in terms of the percentage of positively stained stromal cells and the combinational score of I*P demonstrated the same as result as the stromal intensity score (Fig. 5H, *P* < 0.05).

Taken together, these results demonstrate that the stromal integrin‐α_v_ expression was increased in IM, compared to both normal and oesophagitis tissue and that epithelial integrin‐α_v_ expression was increased in both oesophagitis and IM tissue compared to normal squamous epithelium.

### Barrett's metaplastic cell line is resistant to DCA‐mediated detachment and integrin‐α_v_ internalization

**Figure 6:**
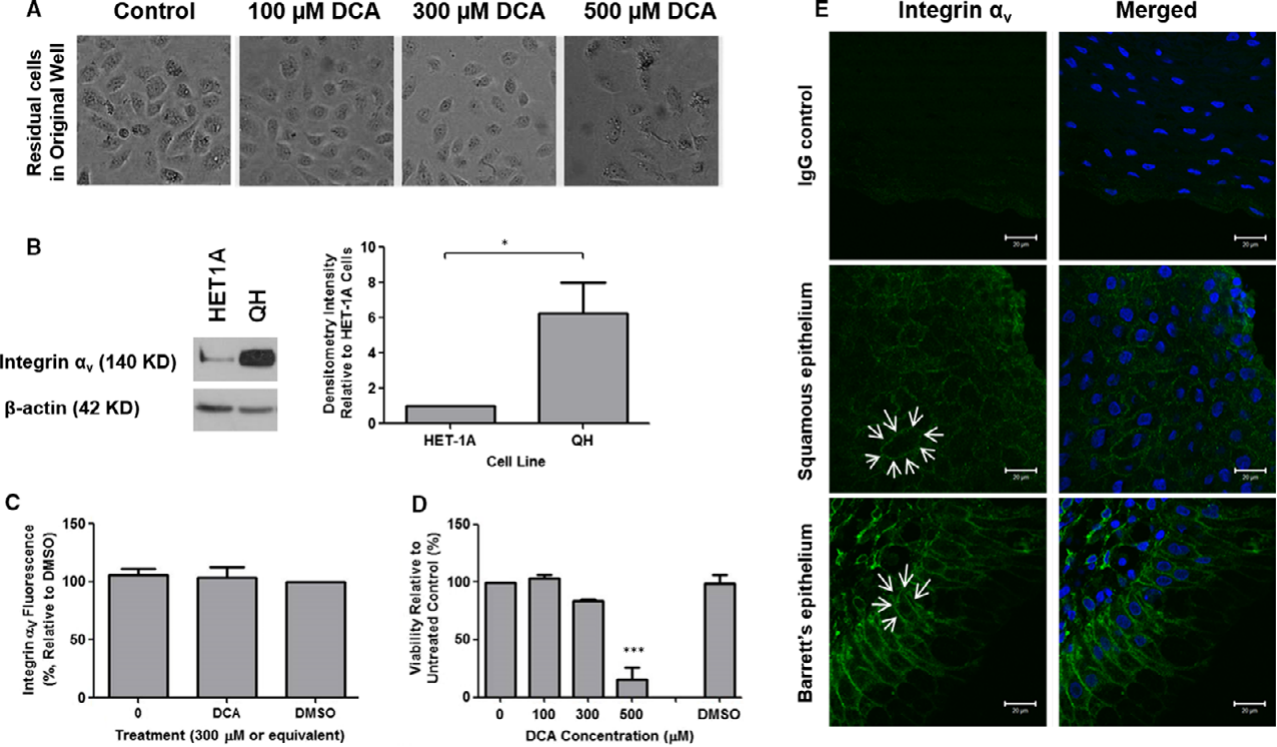
The Barrett's cell line, QH, is resistant to DCA‐mediated detachment and has increased expression of integrin‐α_v_. (**A**) Detachment of QH Barrett's cells was examined in response to 2 hrs DCA exposure by light microscopy. (**B**) Expression of integrin‐α_v_ in HET‐1A and QH cell lines was identified by Western blot and quantified by densitometry analysis (*P < 0.05). (**C**) The reduction in surface expression of integrin‐α_v_ in response DCA stimulation was assessed by flow cytometry. (**D**) Cell viability of QH cells was determined 24 hrs after 2 hrs DCA stimulation (***P < 0.001). (**E**) Immunofluorescent staining of human oesophageal tissue demonstrated increased localization of integrin‐α_v_ at the basolateral side of epithelial cells in the Barrett's epithelium compared to squamous epithelium (arrows). Non‐specific mouse IgG (isotype control) was used as a negative control, and images were acquired using a Zeiss 510 confocal microscope. White bars represent 20 μm.

Metaplastic transformation (i.e. BO) is thought to occur in order to promote a cell type more resistant to the physiological stress experienced by the original tissue. We used a cell line representing BO (QH) to investigate the effects of DCA on detachment, integrin‐α_v_ expression and internalization. Deoxycholic acid did not cause detachment of QH cells (Fig. 6A). Integrin‐α_v_ protein expression was sixfold greater in the cell line representing BO (QH) than in the HET‐1A cell line (Fig. 6B). Deoxycholic acid had no effect on the cell‐surface expression (internalization) of integrin‐α_v_ (Fig. 6C) or total integrin‐αν expression (Fig. S6). Although cell viability was impaired at the highest concentration of DCA studied (500 μM, Fig. 6D), detachment was still not observed (Fig. 6A). In contrast to squamous epithelium, where integrin‐α_v_ is localized diffusely around the cell membrane (Fig. 6E middle panel, arrows), in Barrett's epithelium (Fig. 6E lower panel, arrows), expression was polarized towards the basal side of the epithelial cells. These results suggest that metaplastic transformation is associated with an increase in integrin‐α_v_ expression which could facilitate resistance to reflux‐mediated erosion of oesophageal epithelium.

## Discussion

**Figure 7:**
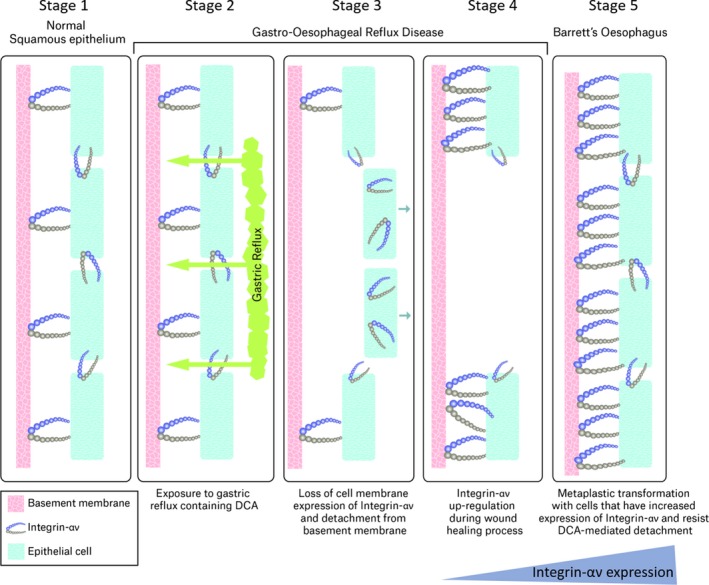
Proposed Mechanism of Action. Exposure of the oesophagus to DCA present in gastric reflux impairs trafficking of integrin‐α_ν_ to the cell membrane through effects on endosomal processing (stage 2), leading to a decrease in cell‐surface integrin expression and detachment of cells from the basement membrane (stage 3). Integrin‐α_ν_ expression is up‐regulated in the resulting ulcerated epithelium facilitating wound healing (stage 4). Re‐epithelialization occurs with metaplastic Barrett's oesophagus cells that have increased expression of integrin‐αν and are resistant to further DCA‐mediated erosion (stage 5).

Low pH and bile acids in oesophageal refluxate are associated with an increased risk of erosive oesophagitis 3 and are independent risk factors for the development of BO, a precursor of oesophageal adenocarcinoma 31. Recent developments in our understanding of oesophageal pathology suggest that alterations in the expression and function of cellular adhesion molecules are involved in benign and metaplastic oesophageal diseases 5, 32, 33. This study reports that oesophageal squamous epithelium exposed to DCA exhibits a reversible reduction in the surface expression of a subset of integrins which promotes cellular detachment and impairs adhesion (Fig. 7). The underlying mechanism of this process is due to impaired endosomal processing of integrins. The *in vitro* findings of reduced surface expression of integrin‐α_v_ in HET‐1A squamous epithelial cells are paralleled in human oesophageal tissue explants transiently (2 hrs) exposed to DCA. These findings are not replicated in QH metaplastic BO cells that have increased expression of integrin‐α_v_ compared to the HET‐1A and are resistant to DCA‐mediated reduction in surface expression of integrin‐α_ν_, potentially offering a survival advantage to this cell type above that of squamous epithelium in the setting of chronic bile reflux. Additionally, the increased expression of integrin‐α_v_ observed in Barrett's metaplastic tissue may provide more robust cellular adhesion, less susceptible to detachment in response to chronic exposure to the bile acid component of reflux observed in these patients. Increased integrin‐α_v_ expression observed in ulcerated tissue in our rat model of reflux‐induced GORD further implicates a role for this protein in the GORD‐BO sequence.

Deoxycholic acid reduced HET1‐A cell adhesion in a dose‐dependent, apoptosis‐independent manner. The fact that DCA reduced surface expression of integrin‐α_5_, α_6_ and α_v_, but not α_3_ and α_4_, indicates a selective process. The reduced cell membrane expression of integrin‐α_v_ was consistent with the reduced adhesion of HET‐1A cells to vitronectin in response to DCA. A similar association can be made between integrin‐α_6_ and the reduced adhesion to laminin. However, the magnitude of the change (compared to vitronectin) was likely smaller for two reasons, firstly, because adhesion to vitronectin was lower than that of other ECM proteins at baseline (data not shown) and, secondly, because a greater number of integrin heterodimers (including integrin‐α_3_) have high affinity for laminin. In support of this hypothesis, adherence to collagen and fibronectin was unaltered, consistent with the constant levels in the surface expression of integrin‐α_3_ and α_4_ 29, 34, 35.

The bile acids predominantly found present in refluxate of patients with oesophagitis and BO comprise of cholic acid, secondary bile acids (including DCA and UDCA) and taurine/glycine conjugates of these bile acids 28. Interestingly, the conjugated bile acid TCDCA and the hydrophilic bile acid UDCA did not exert these effects on integrin‐α_v_ localization. Unconjugated hydrophobic bile acids (e.g. DCA) are more likely to be protonated at neutral pH and as such are capable of traversing cell membranes. This may suggest that the effects of DCA are mediated within the cell membrane or the intracellular compartment rather than at the cell surface. Attempts to reduce the pH of the medium to acidic pH (e.g. pH 4 or 5) resulted in death of the HET‐1A cells, precluding analysis of the effects of acid on integrin expression (data not shown). However, bile reflux in neutral or weakly acidic milieu is the predominant form of reflux in patients receiving acid suppressing medication 36. Animal models of reflux demonstrate that metaplasia and adenocarcinoma are significantly more likely to occur when bile acids are present in oesophageal refluxate 37, 38, 39, 40. In these models, acid suppression was associated with a higher risk of columnar lined oesophagus (the rat equivalent of BO) in the setting of mixed reflux (low pH and bile acid) 40 and acid protected against bile acid‐induced adenocarcinoma in the presence of a tumour promoter 39. There are studies that suggest this may be reflected in human pathophysiology 41, 42.

We next investigated a mechanism for DCA‐mediated reduction in cell‐surface expression of integrin‐α_v_. Rab11 is associated with endosomal recycling through the peri‐nuclear recycling compartment 30. Integrins‐α_5_, α_6_ and α_v_ undergo endocytic recycling through this pathway, while integrins‐α_3_ and α_4_ do not 43, 44. Our findings suggest that DCA is interfering with the endocytic pathway, thus inhibiting recycling of integrin‐α_v_.

We demonstrate that the cell membrane intensity of integrin‐α_v_ was reduced in oesophageal tissue explants after exposure to DCA suggesting that our *in vitro* data are representative of an *in vivo* phenomenon occurring in response to reflux. We propose that the reduction in cell‐surface expression of integrin‐α_v_ in response to DCA leads to reduced adhesive strength within the stratified squamous epithelium. This increases the likelihood of denudation (ulceration) in response to further chemical and mechanical stress. The basal zone of the oesophageal squamous epithelium may be exposed during this process and, in response to chronic reflux, these cells can undergo reactive changes including basal zone thickening and inflammation 45. Expression of integrin‐α_v_ is up‐regulated in the epidermis of chronic wounds and inflammatory tissue and plays a role in wound healing 46, 47. We used an *in vivo* rat model of reflux‐induced GORD to demonstrate increased integrin‐α_v_ expression in ulcerated epithelium compared to non‐ulcerated tissue in the setting of chronic exposure to reflux containing DCA. Healing of these ulcers involves activation of transcription factors and growth factors (EGF, FGF, HGF, VEGF) to facilitate restoration of the epithelium and ECM 48. Integrins play a vital role in co‐ordination of the wound healing process. Integrin‐α_v_ modulates expression of cell‐surface receptors and physically interacts with receptors including Vascular Endothelial growth Factor (VEGF), Matrix Metalloproteinases (MMPs) and Inhibitors of Apoptosis Proteins (IAPs) to facilitate cell proliferation, migration and angiogenesis 46. An increase in expression of integrin‐α_v_ is observed in the latter stages of wound healing and is associated with TGF‐β activation and regulation of ECM deposition 49. Integrin‐α_v_ is also implicated in differentiation and tissue remodelling. The increased expression of integrin‐α_v_ observed in the rat oesophageal ulcerated tissue may facilitate the re‐epithelialization process and attachment to the regenerated ECM.

The diffuse membranous localisation of integrin‐α_v_ in squamous oesophageal epithelium mimics that of alpha integrins previously identified within the oesophagus (e.g. α_2_, α_3_, α_6_ and α_v_) 18, 20. In this location, it is plausible that alterations in integrin expression may also play a role in the development of the dilated intercellular spaces which occur in response to reflux. In contrast to its diffuse localization in squamous epithelium, within BO integrin‐α_v_ expression was polarized towards the basal aspect of the cell suggesting a greater role in adhesion to the basement membrane. However, expression was also observed at the basolateral aspect of the cells in the intercellular spaces suggesting that it may also have a role in intercellular adhesion in Barrett's epithelium.

In addition to our findings of increased expression of integrin‐α_v_ in the BO cell line QH and in BO patient tissue, we demonstrate that exposure of QH cells to DCA did not alter expression of integrin‐α_v_ and QH cells did not detach after DCA stimulation. These findings are consistent with the theory that metaplastic transformation occurs to produce a cell type more resistant to the physiological stress experienced by the original tissue. Consequently, *in vivo* in the setting of erosive oesophagitis driven by chronic exposure to bile acids and low pH, re‐epithelialization with Barrett's cells may be favoured. When BO is established, erosion due to mechanical stress and exposure to bile acids is less likely to occur. Furthermore, in addition to increased adhesive strength, expression of integrin‐α_v_ is associated with increased pro‐survival signalling both *in vitro* and *in vivo* 50. However, the finding that integrin‐α_v_ is highly expressed in BO is also of some clinical concern. High levels of integrin‐α_v_ have been associated with an increased risk of carcinogenesis in solid organ tumours 51 and could therefore play a role in the development of oesophageal adenocarcinoma.

The findings of this study suggest a novel mechanism through which reflux promotes the development of BO. The reduction in cell‐surface expression of integrin‐α_v_ may predispose to denudation/ulceration of squamous oesophageal epithelium. An increase in integrin‐α_v_ expression is observed in oesophageal ulcers, potentially driven by the inflammatory cytokines and growth factors, that have previously been shown to be expressed in ulcerated tissue 52. This increased integrin‐α_v_ expression may facilitate the wound healing process for re‐epithelialisation, but could also promote the metaplastic transformation to an epithelium more resistant to bile acid reflux. When BO is established, erosion due to mechanical stress and exposure to bile acids is less likely to occur. Taken together, the findings of this study suggest a novel mechanism through which bile acids promote both erosive GORD and favour the presence of BO in the GORD oesophagus.

This study highlights that targeting the bile acid component of gastric refluxate should be considered in the clinical management of patients with GORD to prevent erosion of the oesophagus and development of BO.

## Conflicts of interest

The authors confirm that there are no conflicts of interest.

## Author contributions

DP and AMB involved in study design, experimental execution, analysis and interpretation of data, important intellectual contribution, drafting and critical revision of manuscript. JOM and JVR involved in study design and experimental execution for *in vivo* GORD model; JOS involved in case selection and approval for TMAs; RF involved in TMA construction; BD and OSE involved in pathological assessment for TMA construction, re‐evaluation of pathology post‐TMA construction; SF involved in pathology assessment for GORD model; AM involved in re‐evaluation of pathology post‐TMA construction; DD involved in independent grading of TMA staining; DK involved in important intellectual contribution, critical revision of manuscript. AL involved in study design, interpretation of data, important intellectual contribution and critical revision of manuscript

## Supporting information


**Figure S1** High Dose DCA causes detachment of HET1A and a portion of the detached cells re‐adhere.
**Figure S2** Quantification of intensity of membrane staining with integrin α_v_ in tissue explants after DCA treatment.
**Figure S3** UDCA does not affect HET‐1A cell adhesion.
**Figure S4** Neither DCA nor UDCA affect cell viability or induce apoptosis.
**Figure S5** DCA reduced cell surface but not total cellular expression of integrin‐α_v_.
**Figure S6** DCA does not alter total expression of integrin‐α_v_ in Barrett's cells.
