# Supplementary material for: Deoxycholic acid promotes development of gastroesophageal reflux disease and Barrett's oesophagus by modulating integrin‐αv trafficking
Source: J Cell Mol Med. 2017 Sep 22;21(12):3612–25. doi: 10.1111/jcmm.13271 (PMC5706496; doi:10.1111/jcmm.13271)
Supplement: Supplementary file 1 — Figure S1 High Dose DCA causes detachment of HET1A and a portion of the detached cells re‐adhere. Figure S2 Quantification of intensity of membrane staining with integrin αv in tissue explants after DCA treatment. Figure S3 UDCA does not affect HET‐1A cell adhesion. Figure S4 Neither DCA nor UDCA affect cell viability or induce apoptosis. Figure S5 DCA reduced cell surface but not total cellular expression of integrin‐αv. Figure S6 DCA does not alter total expression of integrin‐αv in Barrett's cells. [file JCMM-21-3612-s001.docx]

**Supplementary Data**

Deoxycholic acid promotes development of Gastro-Oesophageal Reflux Disease and Barrett's Oesophagus by modulating integrin-αv trafficking.

Anne Marie Byrne and David O Prichard et al.


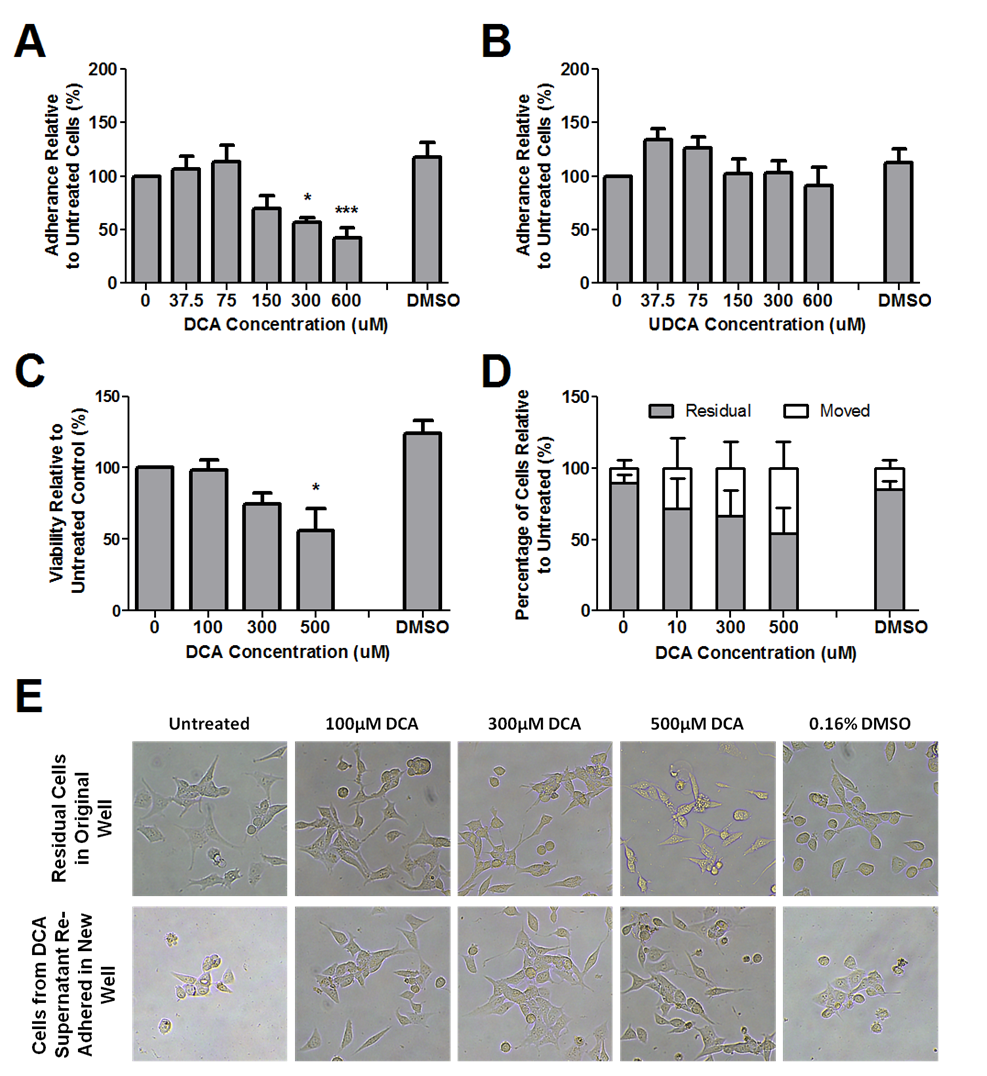


**(A)**

**(B)**


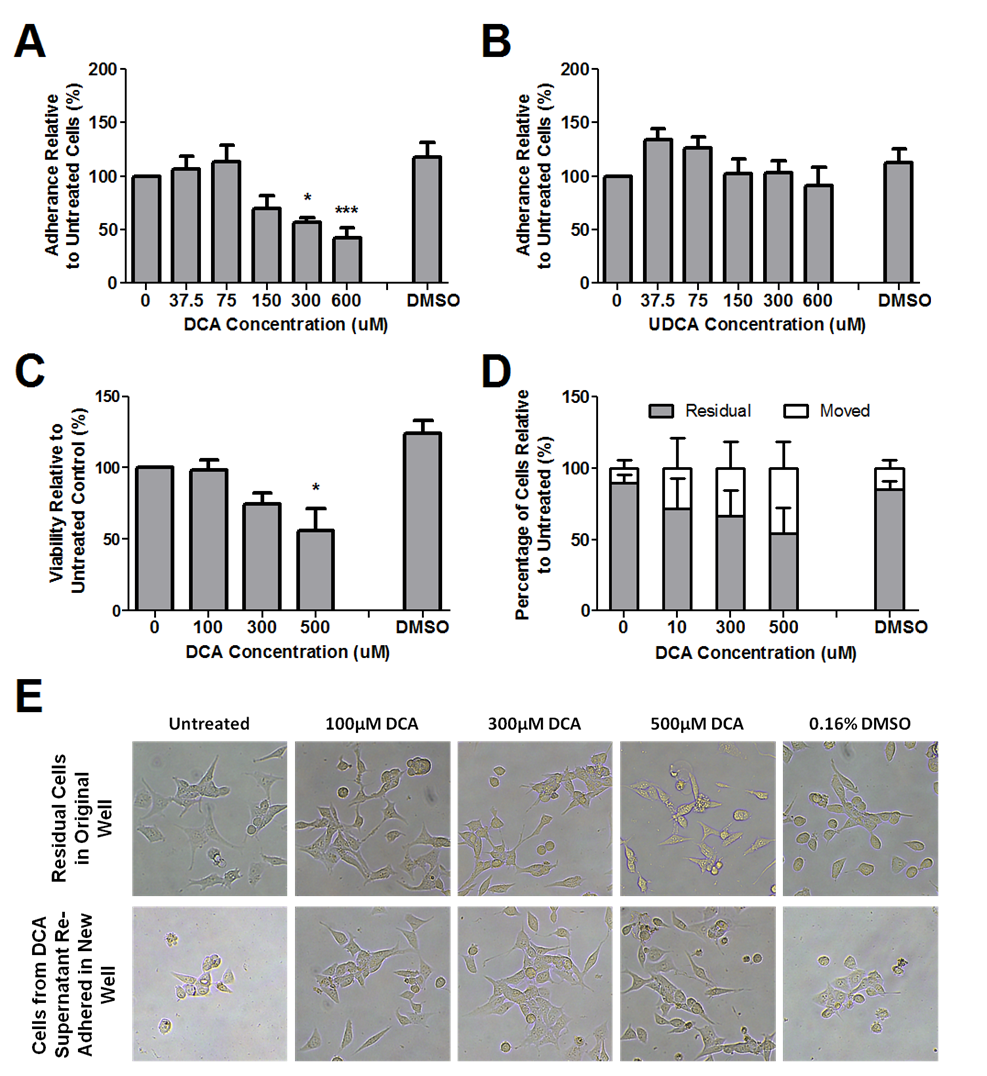

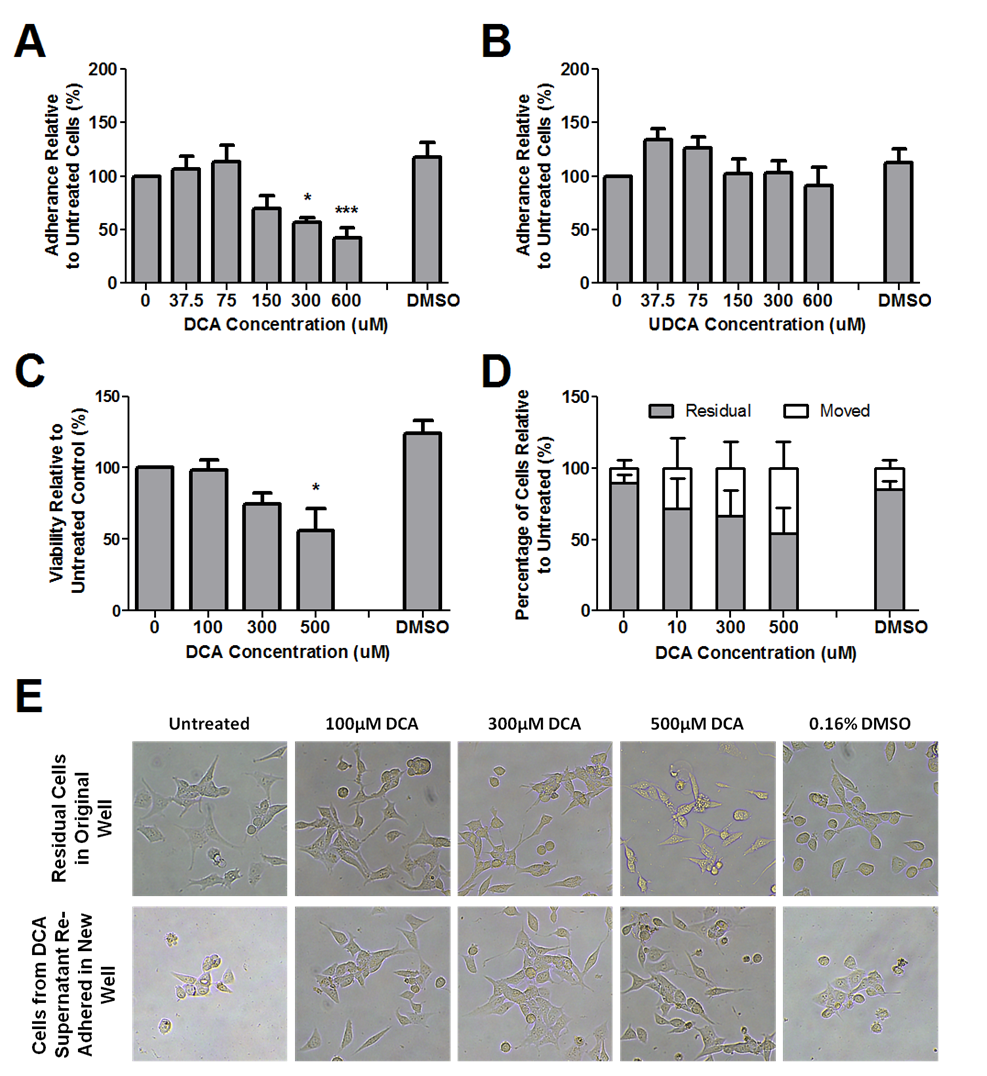

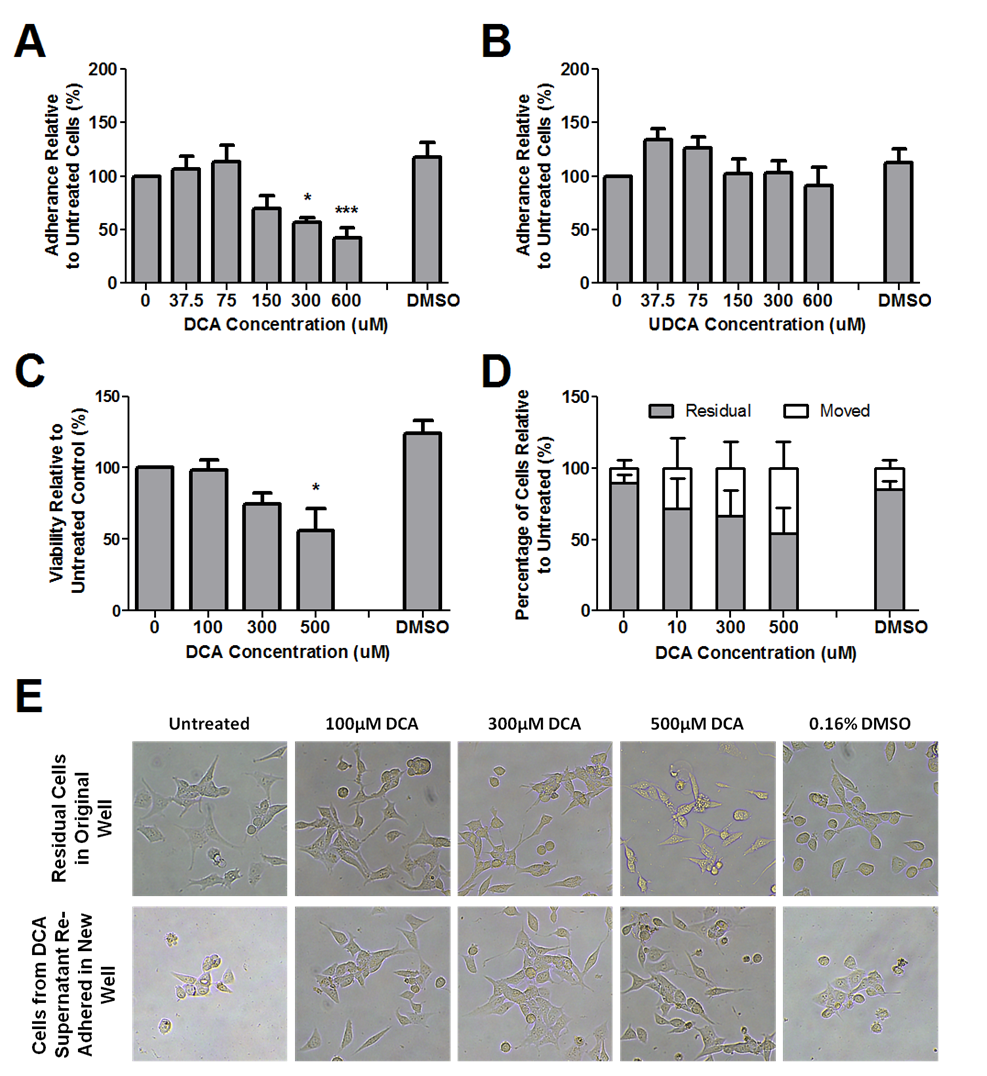

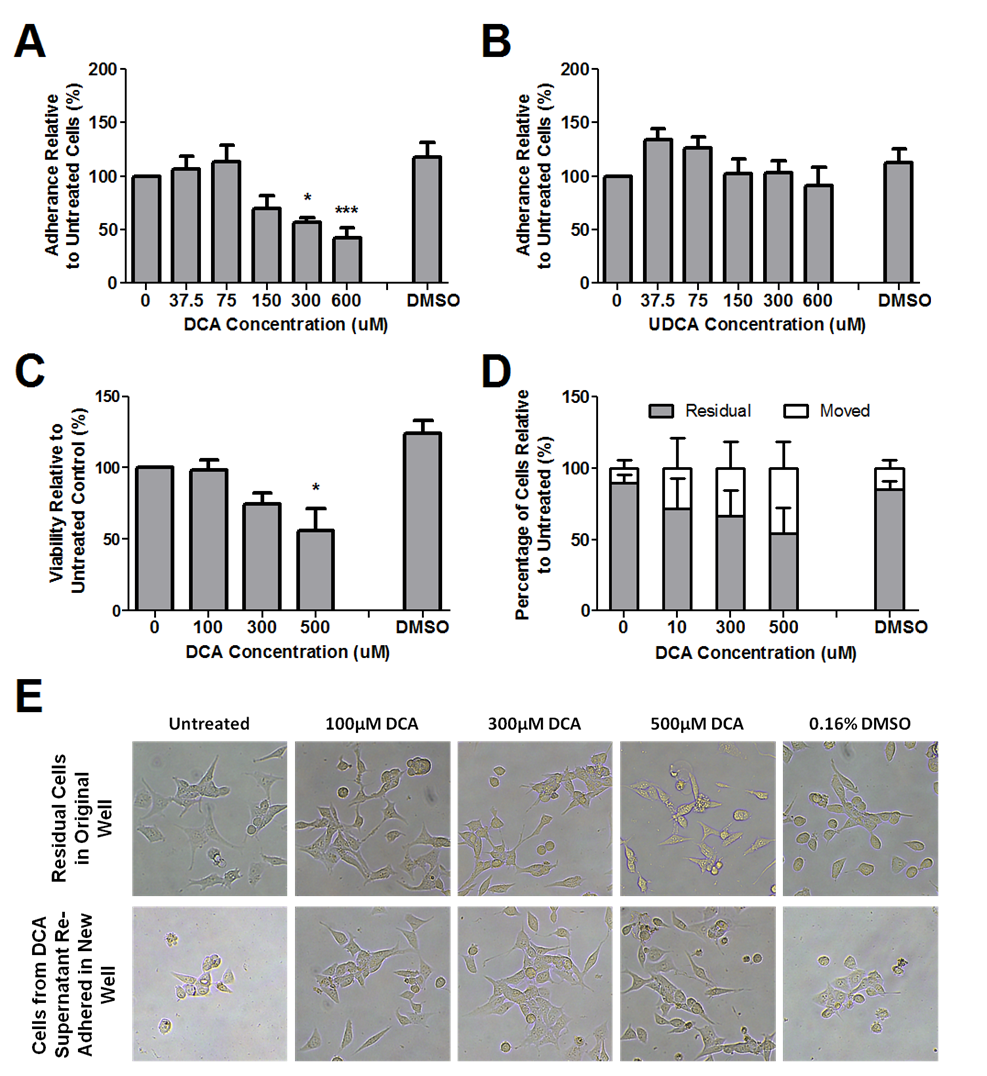


**500μM DCA**

**Control**


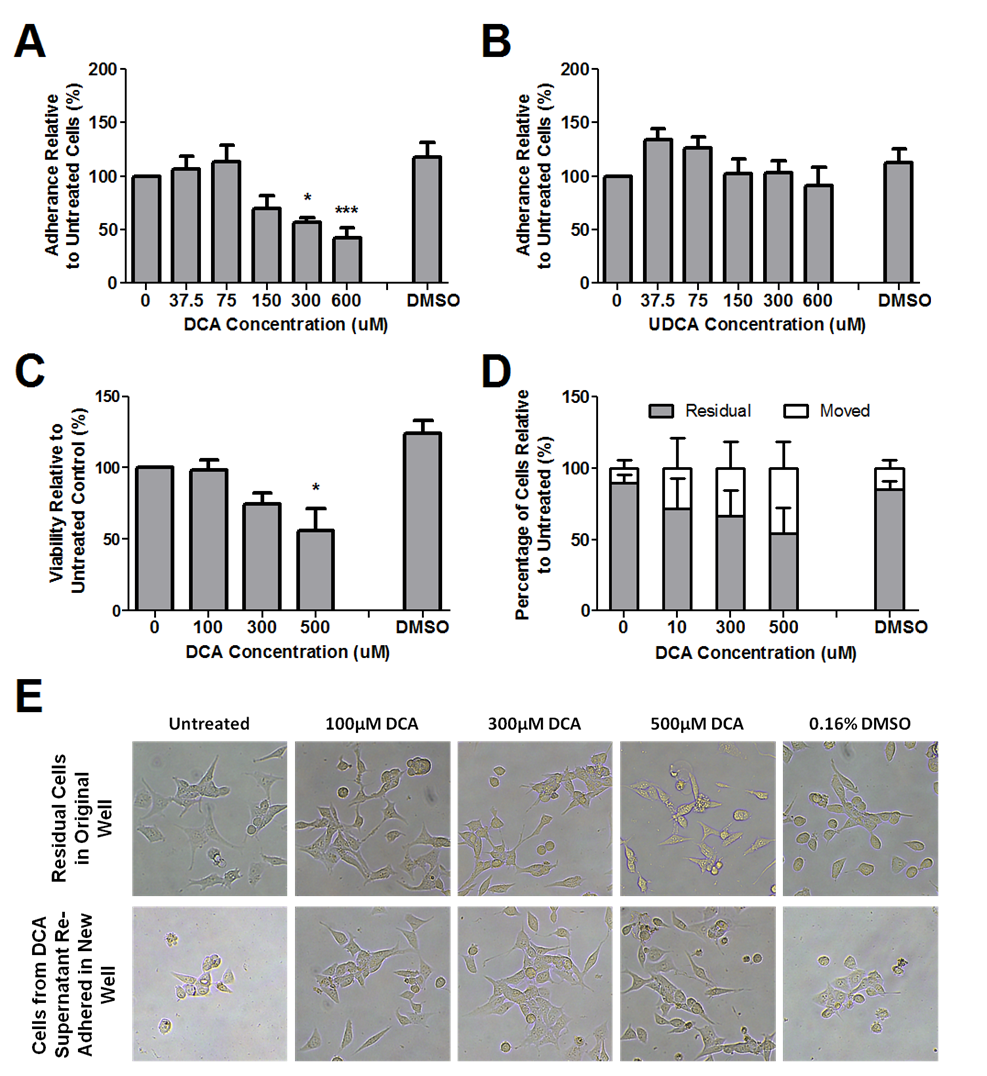


**(C)**

**Supplementary Figure 1. High Dose DCA causes detachment of HET1A and a portion of the detached cells re-adhere. (A)** Adherent HET-1A cells were exposed to DCA (500 µM) or vehicle control for 2h. The residual adherent cells were washed to remove DCA and fresh growth medium added. Detached cells, present in the growth medium used for DCA stimulation, were centrifuged and washed to remove DCA prior to re-seeding in a new culture well. After 24 hours, images were acquired to document re-adherence of the detached cells. (B) Cellular numbers, relative to untreated unmoved cells, were determined by MTT assay. (C) Cell viability was assessed using the MTT assay. Results are presented as mean and SEM for n=3 experiments. * p < 0.05, *** p < 0.001 relative to untreated cells.


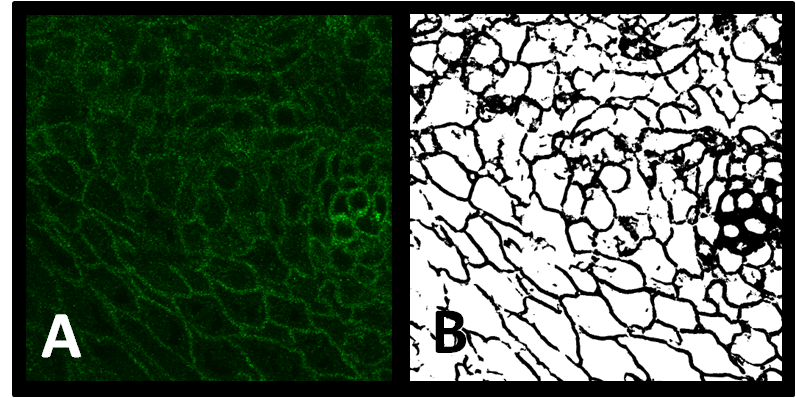

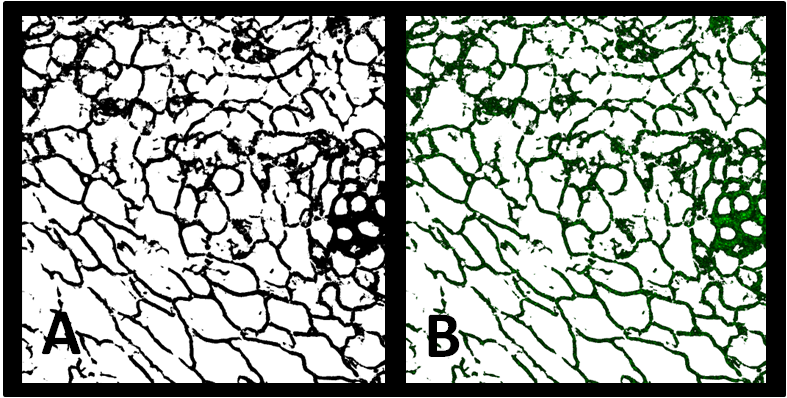

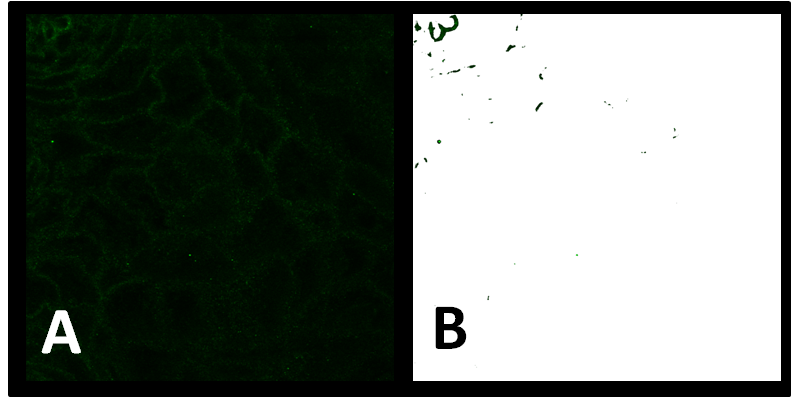


**C**

**A**

**F**

**B**

**D**

**E**

**Supplementary Figure 2. Quantification of Intensity of Membrane Staining with Integrin α_v_ in Tissue Explants after DCA Treatment.**

Tissue explants were acquired, treated, fixed, stained and imaged as described in ‘Methods’. The Trainable Weka Segmentation plugin (available at http://fiji.sc/Trainable_Weka_Segmentation) for image J (US National Institutes of Health, Bethesda, Maryland) was used to define the cell membranes prior to determining membrane intensity. After manually defining cell membranes on a representative image (A), the programme was trained using the following settings:

1. Filters: Gaussian blur, difference of Gaussians, hessian, sobel filter, membrane projections, anisotropic diffusion, structure and entropy
2. Membrane thickness 2.
3. Membrane patch: 19.
4. Sigma min: 2.
5. Sigma max: 16.

The binary image produced was used to generate a black and white binary mask (B).

The generated binary image was used to a mask the background of the original image, isolating the cell membranes . The image and overlay were flattened into a new .tif file (C,D). The pixel intensities of the image (representing fluorescence at a wavelength of 488nm) were then determined. This data was used for comparative purposes.

Where possible, intensity values from the 5 individual images from each explant were acquired and the median intensity for each explant and treatment were determined. Non-parametric analyses were used for comparative statistics.

In some images, it was not possible to identify the cell membrane due to a low signal to noise ratio (E,F). Where fewer than 20,000 (~2%) pixels were identified as membranous, the image was excluded from the analysis.


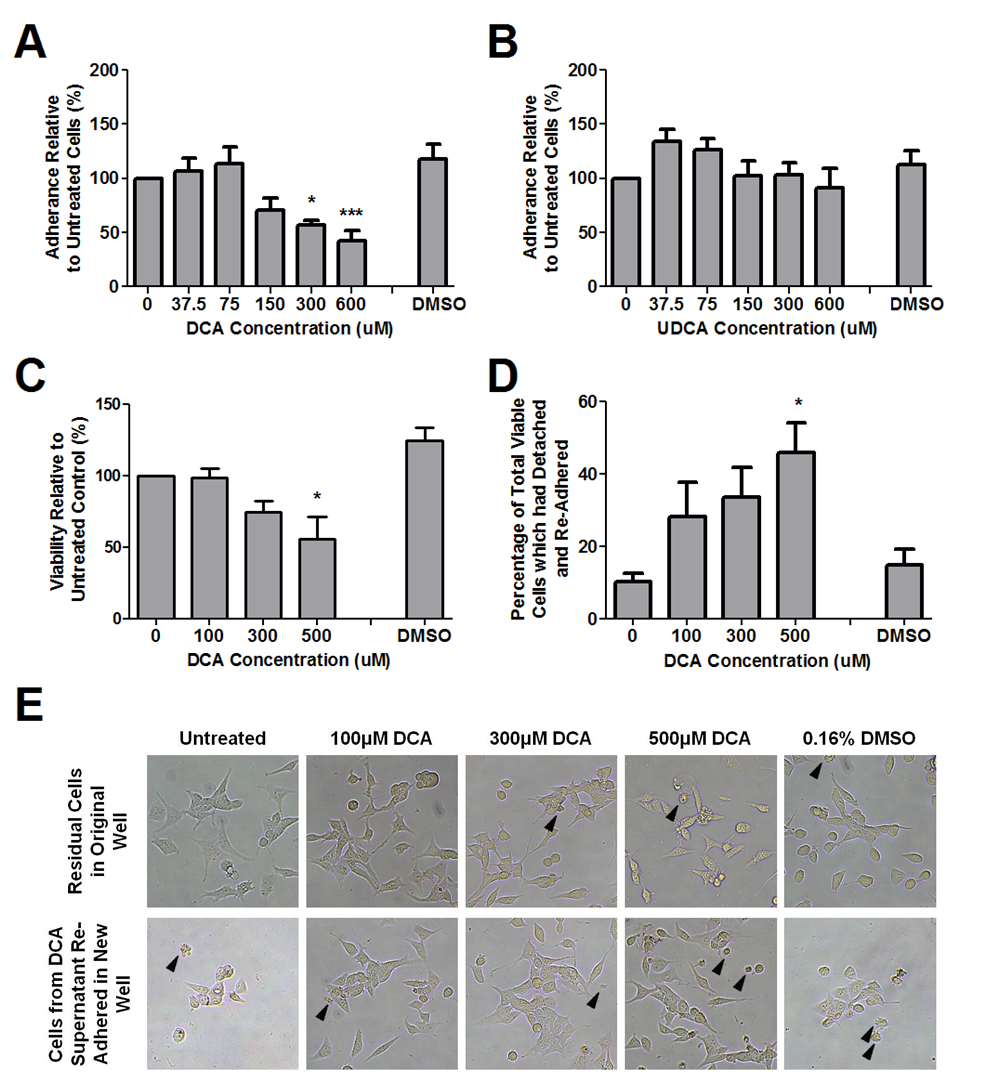


**Supplementary Figure 3. UDCA does not affect HET-1A cell adhesion.** Cultured cells were mechanically detached by gentle scraping and seeded in a fresh well with growth medium containing UDCA for 2 h. UDCA was removed, the cells incubated with Calcein AM and fluorescence determined using a fluorometric plate reader. Adherence was normalised to untreated control. Data are presented as mean ± SEM for n=3 experiments.


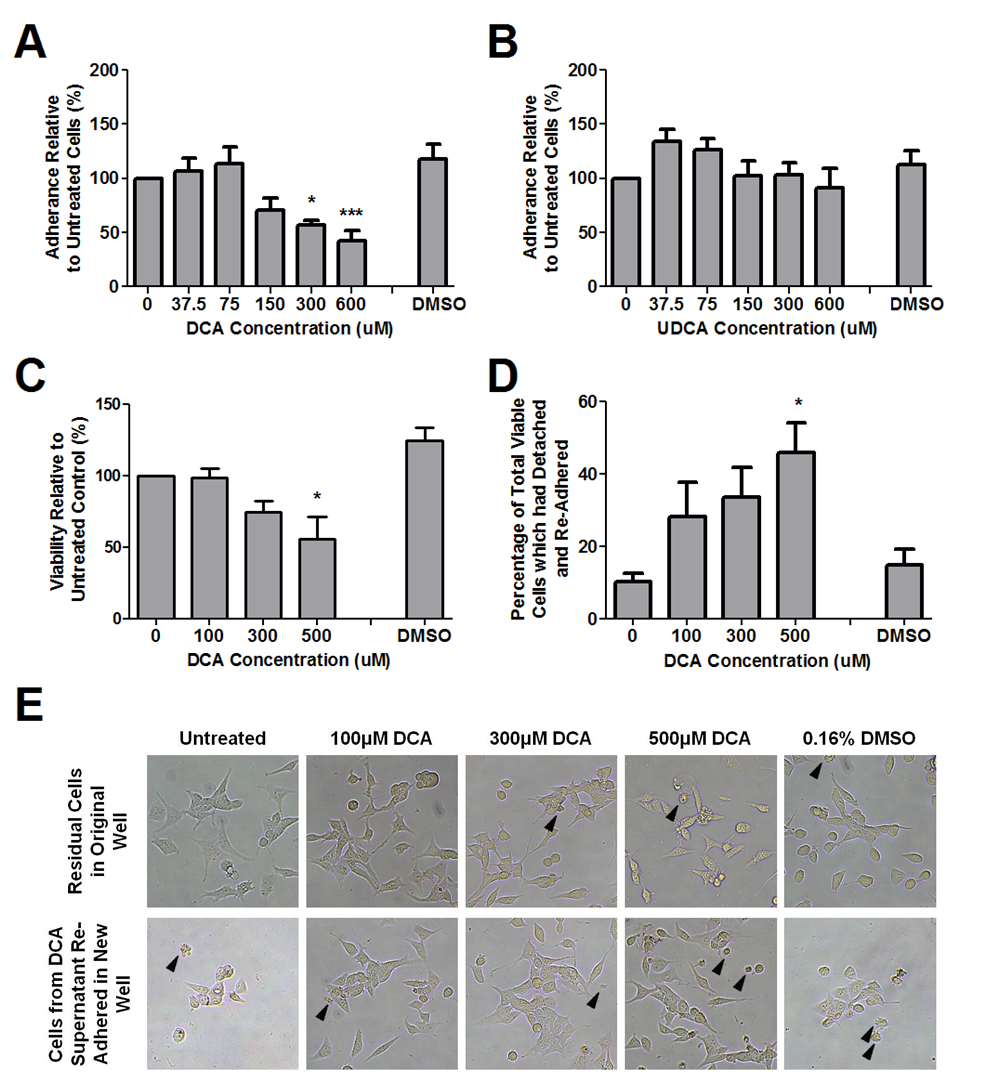


**Supplementary Figure 3. UDCA does not affect HET-1A cell adhesion.** Cultured cells were mechanically detached by gentle scraping and seeded in a fresh well with growth medium containing UDCA for 2h. UDCA was removed, the cells incubated with Calcein AM and fluorescence determined using a fluorometric plate reader. Adherence was normalised to untreated control. Data are presented as mean ± SEM for n=3 experiments

**(B)**

**(A)**


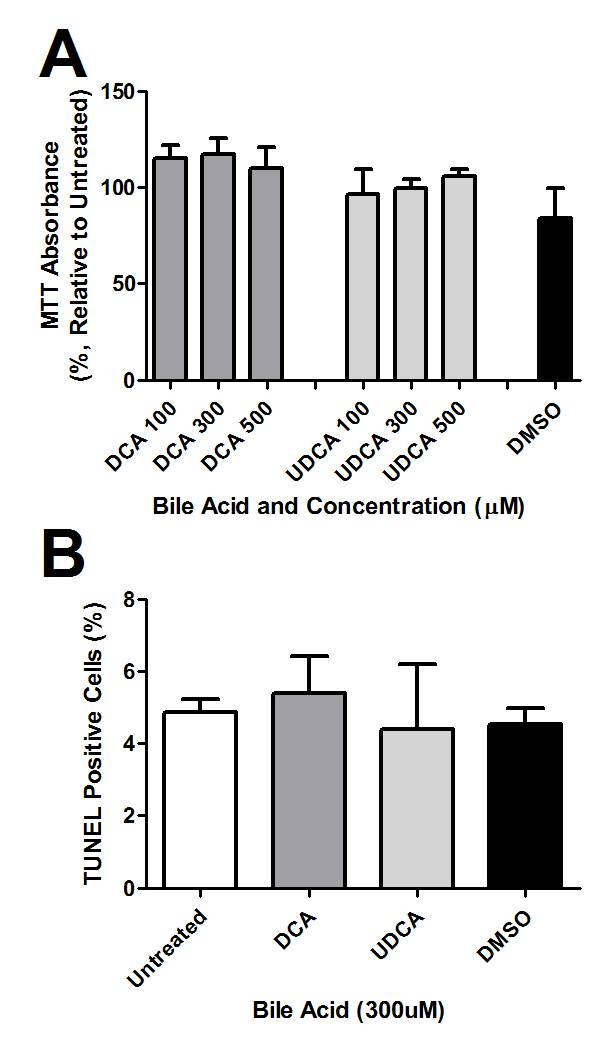


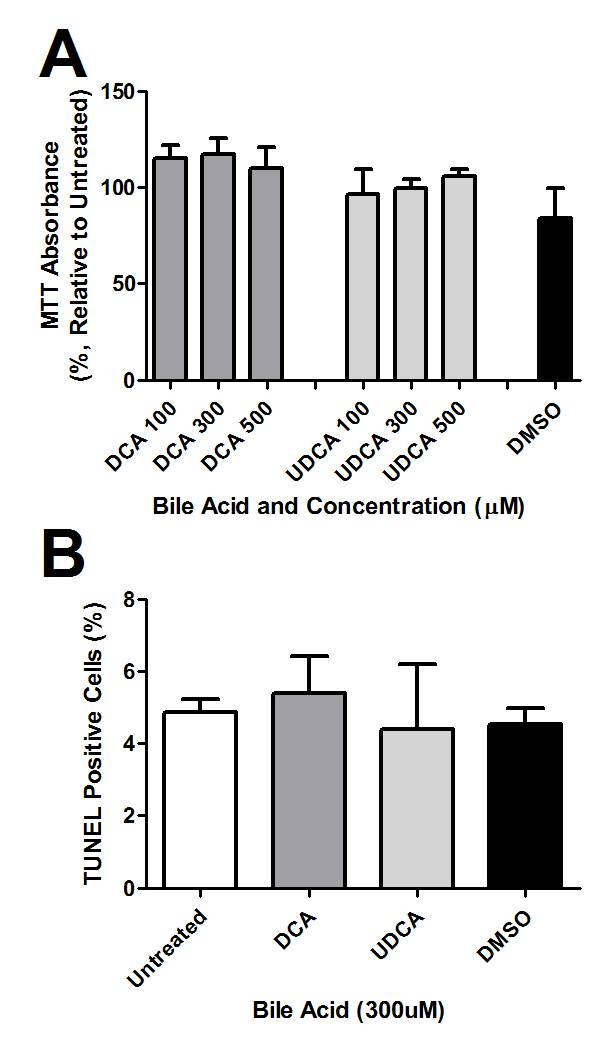


**Supplementary Figure 4. Neither DCA nor UDCA affect cell viability or induce apoptosis.**  HET-1A cells treated with DCA or UDCA for 2 h. After this time cell viability was assessed using an MTT assay (A) and apoptosis was assessed using a Terminal Deoxynucleotidyl Transferase Nick-End Labeling (TUNEL) - Propidium Iodide (PI) assay by flow cytometry (B) Data are presented as mean ± SEM for n=3 experiments.


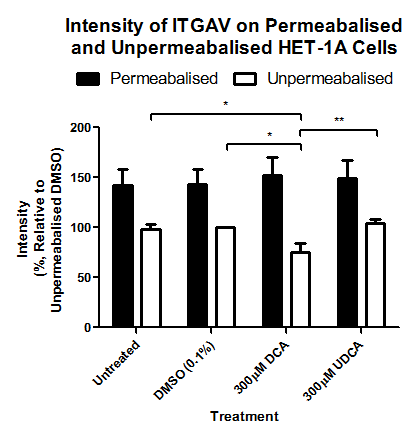


**Supplementary Figure 5. DCA reduced cell surface but not total cellular expression of integrin-α_v._** Adherent HET1-A cells were treated as indicated for 2 h and fixed with 4% Paraformaldehyde. Cells were either permeabilised using 0.1% PBST to monitor effects on intracellular integrin-α_v_ expression or left unpermeabilised to monitor effects on extracellular integrin-α_v_ expression. Cells were stained with anti-integrin-α_v_ antibody and FITC labeled secondary antibody. Images were acquired using High Content Analysis and cell integrin-α_v_ intensity analysed using Investigator software. Data are presented as mean ± SEM for n=3 experiments. (* *P* < .05, ** *P* < .01 respectively).


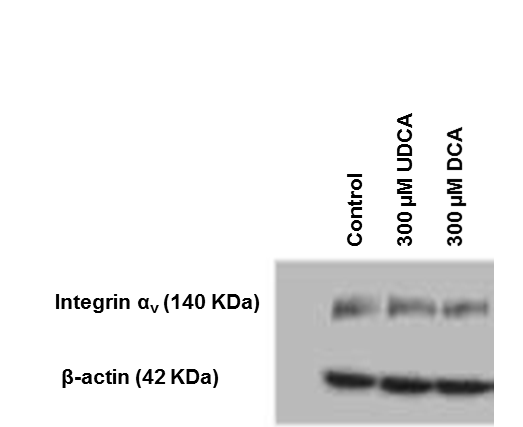


**Supplementary Figure 6. DCA does not alter total expression of integrin-α_v_ in Barrett’s cells.** Adherent QH cells were treated as indicated for 2h. Expression of integrin-α_v_ was assessed by western blot.
